# Clinical implications of proliferation activity in T1 or T2 male gastric cancer patients

**DOI:** 10.1038/emm.2015.79

**Published:** 2015-11-06

**Authors:** Young-Woo Kim, Bang Wool Eom, Myeong-Cherl Kook, Han-Seong Kim, Mi-Kyung Kim, Hai-Li Hwang, Vishal Chandra, Shiv Poojan, Yura Song, Jae-Soo Koh, Chang-Dae Bae, Jungsil Ro, Kyeong-Man Hong

**Affiliations:** 1 Research Institute, National Cancer Center, Goyang, Korea; 2 National Cancer Center Hospital, National Cancer Center, Goyang, Korea; 3 Department of Pathology, Inje University Ilsan Paik Hospital, Goyang, Korea; 4 Department of Pathology, Korea Cancer Center Hospital, Seoul, Korea; 5 Department of Molecular Cell Biology, Sungkyunkwan University School of Medicine, and Samsung Biomedical Research Institute, Suwon, Korea

## Abstract

Proliferation activity has already been established as a prognostic marker or as a marker for anticancer drug sensitivity. In gastric cancer, however, the prognostic significance of proliferation activity is still being debated. Several studies evaluating proliferation activity using Ki-67 have shown controversial results in terms of the relationship between proliferation activity and overall survival (OS) or drug sensitivity in gastric cancer patients. Because cytoskeleton-associated protein 2 (CKAP2) staining has recently been introduced as a marker of proliferation activity, we analyzed 437 gastric cancer tissues through CKAP2 immunohistochemistry, and we evaluated the chromatin CKAP2-positive cell count (CPCC) for proliferation activity. Although the CPCC did not show any significant correlation with OS in the male, female or total number of cases, it did show a significant correlation in the T1 or T2 male patient subgroup, according to log-rank tests (*P*=0.001) and univariate analysis (*P*=0.045). Additionally, multivariate analysis with the Cox proportional hazard regression model showed a significant correlation between the CPCC and OS (*P*=0.039) for the co-variables of age, gender, T stage, N stage, histology, tumor location, tumor size and adjuvant chemotherapy. In male gastric cancer cell lines, faster-growing cancer cells showed higher sensitivity to cisplatin than slow-growing cells. Thus our study indicates that CPCC-measured proliferation activity demonstrates a significantly worse prognosis in T1 or T2 male gastric cancer patients. The CPCC will help to more precisely classify gastric cancer patients and to select excellent candidates for adjuvant chemotherapy, which in turn will facilitate further clinical chemotherapeutic trials.

## Introduction

Because cell proliferation is one of the most vital biological mechanisms in oncogenesis,^1^ cell proliferation activity has been posited as a promising prognostic marker. Whereas the prognostic significance of cell proliferation activity has been well established in various cancers, including breast cancers, meningiomas, gastrointestinal stromal tumors and head and neck cancers,^2, 3, 4, 5^ its utility remains in doubt in other cancers. Particularly in cases of gastric cancer, while some studies have reported a positive correlation between higher proliferation and worse survival,^6^ others have demonstrated no such relationship.^7, 8, 9^ Recently, an inverse correlation was reported in a relatively large (245 cases) cohort.^10^ Therefore, additional studies are required to clarify the prognostic significance of proliferation activity in gastric cancer.

High proliferation activity has been related to greater sensitivity to anticancer drugs.^11^ In breast cancer, for example, proliferation has been recognized as a reliable predictor of the response to adjuvant^12, 13^ and neoadjuvant chemotherapy.^14, 15, 16^ In gastric cancer, however, the correlation between proliferation activity and anticancer drug sensitivity remains unclear. If such a correlation is also observed in gastric cancer, the measurement of proliferation activity for that disease could have therapeutic implications.

Ki-67 and mitotic counts have been the most widely employed tools used to evaluate proliferation activity in various cancers, including gastric cancer. Cytoskeleton-associated protein 2 (CKAP2) has been recently established as a new mitotic marker, with chromatin CKAP2-positive cells being identified as mitotic cells^17^; indeed, there is a strong correlation between the CKAP2-positive cell count (CPCC) and the mitotic figure count.^18^ The prognostic significance of the CPCC has also been demonstrated in breast cancer, for which it was equivalent to or better than the significance of the mitotic activity index,^19^ one of the most widely accepted proliferation activity measurements in breast cancer.^20^ In the present study, to evaluate the prognostic significance of proliferation activity in gastric cancer, CKAP2 immunohistochemical staining was performed on 437 gastric cancer tissues, and the correlation between the CPCC and overall survival (OS) was evaluated.

## Materials and methods

### Patients, specimens and cell lines

Cases of gastric cancer patients who underwent curative resection at the National Cancer Center between 2002 and 2003 were accrued, and microarrays were created from paraffin-embedded tissues from 521 gastric cancer patients. Access to and usage of the patients' clinical information and the relevant archival tissues were approved by the Institutional Review Board of the National Cancer Center, which waived the need for informed consent. The human male gastric cancer cell lines Kato III, SNU-484, SNU-601 and SNU-668 were obtained from the Korean Cell Line Bank (http://cellbank.snu.ac.kr) and cultured in RPMI-1640 culture media (Thermo Fisher Scientific Hyclone, Logan, UT, USA) containing 10% fetal bovine serum (Thermo Fisher Scientific Hyclone) at 37 °C under 5% CO_2_.

### Immunohistochemistry

Immunohistochemical staining was performed using the Ultravision LP Detection kit (Thermo Fisher Scientific Inc., Fremont, CA, USA), as previously reported, for the same CKAP2 antibody.^12^ Briefly, after deparaffinization of the formalin-fixed, paraffin-embedded tissues, antigen was retrieved in 10 mM citrate buffer, pH 6.0, containing 0.05% Tween 20. The tissues were sequentially treated with 3% hydrogen peroxide and Ultra V block solution for 15 min each. After being incubated for 1 h at room temperature with anti-CKAP2 antibody, the slides were washed in Tris-buffered saline with Tween 20 (TBST), incubated with primary antibody enhancer for 10 min and exposed to horseradish peroxidase-conjugated secondary antibody for 15 min. After re-washing in TBST, the tissue slides were incubated with diaminobenzidine chromogen (Scytek Laboratories Inc., Logan, UT, USA) and counterstained with Mayer's hematoxylin (Dako Cytomation, Glostrup, Denmark).

### Chromatin CPCC counts

In the CPCC determination, the number of chromosomal CKAP2-positive cells under one × 200 power field (instead of 10 × 400 power fields) was counted because of the limited number of microscopic fields in the tissue microarrays. Strongly to moderately stained chromatin-positive cells were included in the count. Cores containing <50% tumor area were excluded, and finally, 437 of the original 521 cases were statistically analyzed.

### Cell doubling time (DT) assay

Kato III, SNU-484, SNU 601 and SNU-668 cells (1.5 × 10^3^ cells per well) were seeded in 24-well plates for 24 or 48 h of incubation, in six-well plates for 72 or 96 h of incubation or in 25-mm flasks for 120 or 144 h of incubation. The cell number was counted after trypsinization into a single cell suspension. The cell DT was calculated using the software tool introduced by Roth (http://www.doubling-time.com/compute.php) according to the following formula: DT=(*t*−*t*_0_) log2/(log*N*−log*N*_0_), where *t* and *t*_0_ are the time points at counting and initial plating, respectively, and *N* and *N*_0_, respectively, are the cell numbers at those same time points. Experiments were performed in triplicate, and the results were recorded as the mean DT±s.d.

### Cell growth after cisplatin treatment

Cells (Kato III, SN-484, SNU 601, SNU-668) were plated at 3–7 × 10^4^ cells per well in 24-well plates. After 24 h of incubation, the cells were incubated for a further 72 h with cisplatin, an anticancer agent, at various concentrations (0, 1, 2.5, 5, 10 and 20 μM). Subsequently, the cells were fixed with 4% formaldehyde at room temperature for 5 min and then stained with crystal violet (Sigma, St Louis, MO, USA) for 10 min. Upon completion of the washing and drying procedures, the cells were solubilized with 1% sodium dodecyl sulfate, and the absorbance at 595 nm was measured. The data were normalized to the untreated controls. Experiments were performed in three independent assays, each in triplicate. The dose–response curve was plotted using a non-linear regression model, and the IC_50_ was determined from the fitted curves using GraphPad Prism, version 5 (GraphPad Software Inc., San Diego, CA, USA).

### Statistical analysis

The correlation between the CPCC and the clinicopathological parameters was analyzed with a two-sided Wilcoxon rank-sum test, and a *P*-value <0.05 was considered to be statistically significant. To estimate the prognostic significance of the CPCC, total cases were equally divided into three groups based on the CPCC: group 1, ⩽6 (*N*=156); group 2, 7–17 (*N*=151); and group 3, ⩾18 (*N*=130). OS was defined as the time from radical surgical resection to either the most recent follow-up date or until death. In Kaplan–Meier plots of the correlation with OS, the prognostic significance was analyzed using a log-rank test. Multivariate analyses were performed using Cox's proportional hazard regression model (hazard ratios with 95% confidence intervals (CIs)) after adjusting for age, gender, T stage, N stage, metastasis, histology, tumor location, tumor size and adjuvant chemotherapy. The linear trend was calculated using the median value for each exposure parameter as a continuous variable. The correlation between the DT and the sensitivity to cisplatin was tested with Spearman's correlation test. The statistical analyses were performed with GraphPad Prism or STATA version 13 (StataCorp LP, College Station, TX, USA).

## Results

### Immunostaining patterns of CKAP2 in gastric cancer tissues

**Figure 1:**
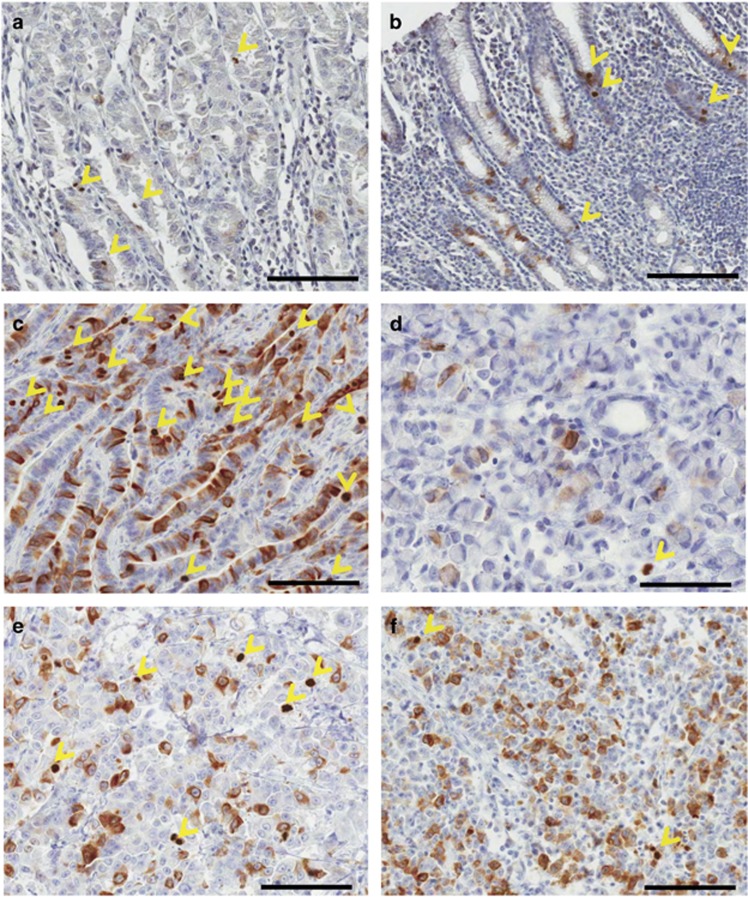
CKAP2 immunohistochemical staining pattern in gastric cancer tissues. (**a**) Normal stomach body adjacent to cancer cells. (**b**) Normal stomach antrum adjacent to cancer cells. (**c**) Intestinal type and well-differentiated adenocarcinoma. (**d**) Signet-ring cell carcinoma. (**e**) Poorly differentiated adenocarcinoma. (**f**) Poorly differentiated adenocarcinoma with a relatively high number of cytoplasmic CKAP2-positive cells. The yellow arrow heads indicate chromatin CKAP2-positive cells. Each bar represents 100 μm.

Immunohistochemical staining of normal gastric tissues adjacent to cancer cells revealed few chromatin CKAP2-positive cells and a low CPCC (Figures 1a and b). In the gastric cancer tissues, the CPCC varied according to the pathological type: the level by World Health Organizaation classification was relatively higher in well-differentiated adenocarcinomas (Figure 1c) than in signet-ring cell carcinomas (Figure 1d). The level by Lauren classification was relatively higher in the intestinal type (Figure 1a) than in the diffuse type (Figures 1e and f). In several cases of low CPCC, many cytoplasmic CKAP2-positive cells that might have been in the G2 phase and had not progressed to the mitotic phase (possibly G2-arrested cells) were shown (Figure 1f), but their significance was not evaluated in the present study.

### Correlation between the CPCC and clinicopathological characteristics

The clinicopathological characteristics of the 437 gastric cancer patients are provided in Table 1. The CPCC distribution was 0–170, with a median value of 11 (quartile range, 4–20). Wilcoxon rank-sum tests showed that the median was higher for males than for females (*P*=0.002, Table 1) and also higher for the older age groups than for the younger age groups (<50 years) (*P*=0.003–0.011, Table 1). The CPCC distribution was also significantly higher in cases with a higher T classification or depth of invasion (Table 1). All CPCCs differed markedly by pathological type: the level was significantly lower in signet-ring cell carcinoma than in well-differentiated or moderately differentiated adenocarcinoma (*P*<0.001), and it was lower in the diffuse type than in the intestinal type (*P*<0.001, Table 1). However, the CPCC did not differ significantly by the presence of metastasis, lymph node metastasis or tumor size (Table 1).

### No significant correlation between the CPCC and OS in overall gastric cancer cases

**Figure 2:**
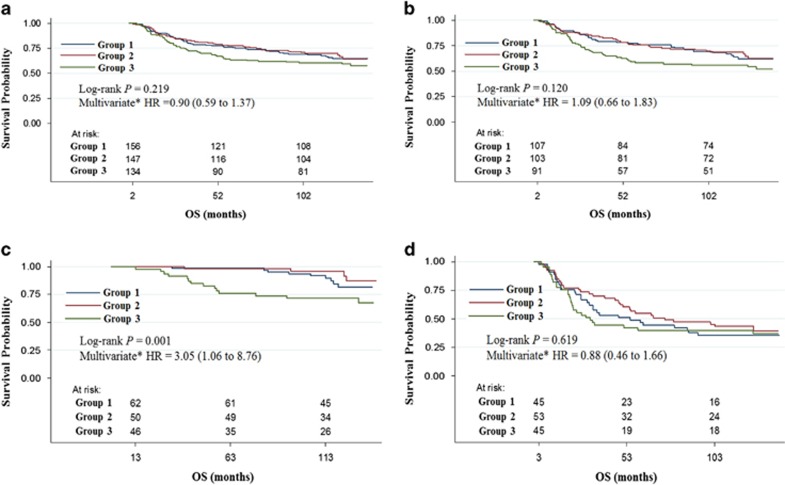
Correlation between the CPCC and OS. Kaplan–Meier plots of the CPCCs for the (**a**) total, (**b**) male, (**c**) T1 or T2 male and (**d**) T3 or T4 male gastric cancer cases are shown. The *P*-values were determined through a log-rank test. The HRs and 95% CIs of the CPCC group 3 (highest tertile, CPCC⩾18) are compared with the CPCC group 1 (lowest tertile, CPCC⩽6) through multivariate analyses, as shown. The numbers at risk are also shown. CPCC, chromatin CKAP2-positive cell count; *x* axis, OS in months; *y* axis, survival probability.

The patients were allocated into three subgroups based on their CPCC values (group 1, 0–6; group 2, 7–17; group 3, ⩾18). No significant correlation with OS was found for the total number of gastric cancer cases, according to the log-rank test (*P*=0.219, Figure 2a), univariate analysis (*P*=0.188, Table 2) or multivariate analysis using the co-variables of age, gender, T classification, N classification, metastasis, histological type, tumor location, tumor size and adjuvant chemotherapy (*P*=0.612, Table 2).

### Correlation between the CPCC and OS in gastric cancer subgroups

To investigate the prognostic significance of the CPCC in subgroups of gastric cancer, the possible subgroups were deduced based on the differential CPCC levels among the clinical parameters. Because there was a significant difference in the CPCC between male and female patients (*P*=0.002, Table 1), they were analyzed separately. However, in a log-rank test, the CPCC showed no significant correlation with OS in either the male (*P*=0.120, Figure 2b) or female patients (*P*=0.697, Supplementary Figure S1). Correspondingly, the univariate and multivariate analyses showed no significance (Table 3). However, worse OS among CPCC group 3 was more marked among the male patients than among the total cohort.

Additionally, because the CPCCs were significantly higher in the advanced T classification cases, T subgroups were formed. Male patients, given the marginality of the prognostic significance among them, were analyzed separately in terms of the T classification. We found a significant correlation between CPCC and OS in T1 or T2 male patients (*P*=0.001, Figure 2c) but not in T3 or T4 male patients (*P*=0.619, Figure 2d). The correlation was also significant according to the univariate analysis (*P*=0.045, Table 3) and multivariate analysis using the co-variables of age, gender, T classification, N classification, histological type, tumor location, tumor size and adjuvant chemotherapy (*P*=0.039, Table 3). In the relapse-free survival analysis, CPCC also demonstrated a significant correlation in T1 or T2 male patients (*P*=0.010, log-rank test).

### Correlation of cell DT with IC_50_ for cisplatin

**Figure 3:**
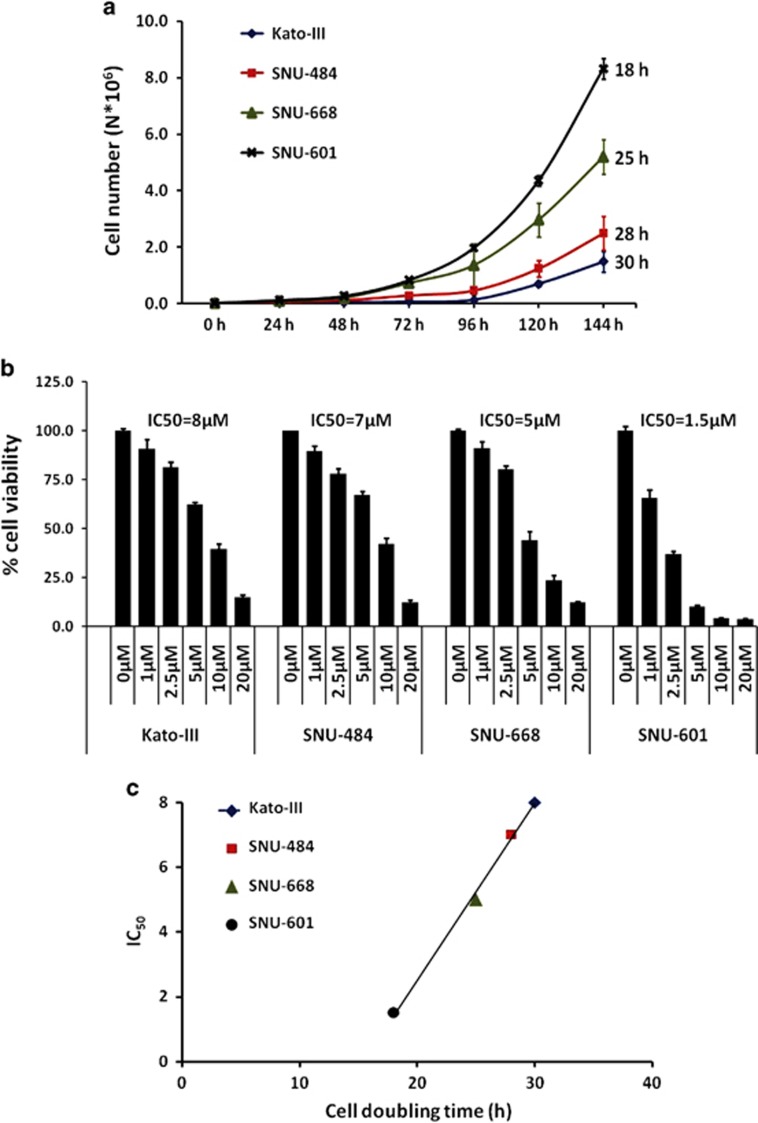
Correlation between cell doubling time and sensitivity to cisplatin in male gastric cancer cells. (**a**) Cell growth as measured by counting cell number (mean±s.e.m., *N*=3) at the indicated time points for the following male gastric cancer cell lines: Kato III, SNU-484, SNU 601, and SNU-668. The cell doubling time (calculated by the exponential regression method) for each cell is indicated at the end of the graph line. (**b**) Sensitivity to cisplatin, as measured by crystal violet after the treatment with various concentrations of cisplatin, is indicated on the *x* axis for 72 h. On the *y* axis, the percentage of viable cells (mean±s.e.m., *N*=5) after calculating the ratios between the treated and the control cells is shown. The IC_50_ is shown for each cell line. (**c**) Positive correlation between shorter cell doubling time and higher sensitivity to cisplatin (*R*=0.999, *P*=0.083). On the *x* axis is the cell doubling time; on the *y* axis, the IC_50_.

In the assessment of DT for the four male gastric cancer cell lines, the results varied: Kato III, 30 h; SNU-484, 28 h; SNU-668, 25 h; and SNU-601, 18 h (Figure 3a). In the evaluation of IC_50_ for cisplatin, the results for Kato III, SNU-484, SNU-668 and SNU 601 were 8, 7, 5 and 1.5 μM, respectively (Figure 3b). Although not statistically significant, a positive correlation between DT and IC_50_ was observed (*R*=0.999, *P*=0.083 by Spearman's correlation test, Figure 3c), suggesting a relationship between faster growth rate and greater sensitivity to cisplatin.

## Discussion

To clarify the prognostic significance of proliferation activity in gastric cancer, the CPCC was determined in 437 gastric cancer cases using CKAP2 immunohistochemical staining. Although the prognostic value of the CPCC among the total gastric cancer patients was not significant (*P*=0.219), it was significant in the T1 or T2 male patient subgroup (*P*=0.001). Multivariate analysis revealed that the CPCC showed prognostic significance (*P*=0.039) for the co-variables of age, gender, T classification, N classification, histological type, tumor location, tumor size and adjuvant chemotherapy. Our results suggest that proliferation activity is a significant prognostic factor in T1 or T2 male gastric cancer patients.

The prognostic significance of proliferation activity in gastric cancer has been controversial. Most relevant studies have found no such prognostic significance in gastric cancer,^7, 8, 9^ whereas one investigation reported a positive correlation between proliferation activity and worse OS.^6^ In contrast to both of these results (negative and positive), we observed a positive correlation only in the T1 or T2 male patient subgroup. Another study found an inverse correlation between proliferation activity and worse OS,^10^ which is inconsistent with our results; however, decreased proliferation activity in diffuse-type or signet-ring cell carcinomas was also reported in that study,^10^ which is consistent with our data. Although our results could not resolve all ambiguities surrounding the question of the prognostic significance of proliferation activity in gastric cancer, the results did demonstrate definitive significance for a specific subgroup.

To date, it is unclear how the subgroup specificity of the prognostic significance of proliferation activity in gastric cancer can be explained. Such subgroup specificities have already been reported for other cancers, for example, breast cancer,^19, 20, 21, 22, 23^ but not in gastric cancer. In the present study, the cancer tissues of the male patients showed a significantly higher CPCC than those of the female patients, which could be related to biological or hormonal differences. Indeed, the protective roles of female hormones, in terms of gastric cancer, have already been delineated.^24^ In addition to the gender difference, our data suggested that invasion depth is an important confounding factor in terms of the prognostic significance of proliferation activity in gastric cancer. Because the CPCC was higher in advanced T cancers, and because the prognostic significance of the CPCC was shown only in T1 or T2 male patients but not in T3 or T4 male patients, the growth rate might be important to patient survival only in early T gastric cancer, whereas cancer cell invasion and escape from anatomical barriers might be more important in cases of advanced T disease. Accordingly, the present study's analysis of relevant factors, such as gender and T classification, led to the identification of a specific gastric cancer subgroup for which the CPCC or proliferation activity has prognostic significance. In fact, successful targeted therapy for *HER2*-amplified gastric cancer patients strongly suggests the existence of therapeutically meaningful gastric cancer subgroups,^25^ even if the molecular classification of that malignancy is still in its infancy. Significantly, a recent molecular analysis indicated that 37% of gastric cancer cases can be classified based on the copy gains of five drug-target genes,^26^ which again strongly suggests the existence of gastric cancer subgroups.

The identification of the T1 or T2 male subgroup in gastric cancer, as based on the CPCC or proliferation activity, might have clinical implications for chemotherapeutic intervention, given that highly proliferating cells are generally more susceptible to chemotherapy.^27^The importance of proliferation activity in the setting of breast cancer chemotherapy intervention has already been indicated, specifically for stronger responses to chemotherapy in cases of higher proliferation indices.^28, 29^ In the present study, we showed a positive correlation between shorter DT and higher sensitivity to cisplatin in male gastric cancer cell lines. If highly proliferative gastric cancer cells are more susceptible to chemotherapy, T1 or T2 male gastric cancer patients with highly proliferative cancer cells might be better candidates for chemotherapy. Further chemotherapeutic intervention studies of this subgroup could reveal additional clinical implications.

Although we did not validate the prognostic significance of the CPCC in T1 and T2 male patients in an independent set of gastric cancer cases, we did estimate the prognostic significance among the largest gastric cancer cohort (437) studied thus far (the relevant previous studies have all studied approximately 200 cases^6, 7, 9, 10^).

In conclusion, we identified the prognostic significance of the CPCC in a subgroup of T1 or T2 male gastric cancer patients, thus making a constructive contribution to the greater prognosis-based classification of gastric cancer. Furthermore, our results suggest that a T1 or T2 male gastric cancer patient subgroup with high proliferation activity is an excellent candidate for adjuvant chemotherapy, the data from which will facilitate additional clinical chemotherapeutic trials with this gastric cancer subgroup.

## Figures and Tables

**Table 1:** Correlation between the CPCC and clinicopathological characteristics of the study population

*Variable*	*Group*	N	*Median CPCC (quartile range)*	P
Gender	Male	301	12 (6, 21)	
	Female	136	8 (2, 17)	**0.002**
Age, years	<50	119	8 (2, 17)	
	50–59	123	13 (5.5, 20)	**0.003**
	60–69	146	11 (5, 20.8)	**0.009**
	⩾70	49	11 (5, 23)	**0.011**
Histologic type (WHO)	WD or MD	166	13 (6, 21.8)	
	PD	178	10 (4, 18.8)	0.071
	SRC	68	5.5 (2, 12)	**<0.001**
	Others	25	18 (6.75, 44.75)	0.068
Lauren classification	Intestinal	207	12 (6, 21)	
	Diffuse	151	6 (2, 14.8)	**<0.001**
	Mixed	36	10 (5, 19.8)	0.343
	Unknown	43	14.5 (9.3, 32.5)	**0.010**
Depth of invasion	T1	183	9 (3.5, 17)	
	T2	44	15 (4, 28)	**0.035**
	T3	83	11 (5, 18)	0.200
	T4	127	12 (6, 22)	**0.038**
LN metastasis	N0	219	10 (4, 18.5)	
	N1	50	12.5 (6, 22)	0.351
	N2	58	11 (5, 17)	0.586
	N3	110	11 (5, 23.5)	0.340
Metastasis	No	411	11 (4, 20)	
	Yes	26	8.5 (6, 14.5)	0.840
Tumor location	Middle	81	8 (4, 17)	
	Upper	53	9 (2, 16)	0.624
	Lower	263	11 (5, 21)	0.050
	Overlapping	40	13 (4.8, 22)	0.153
Tumor size	1 (⩽3.0 cm)	99	10 (3, 18)	
	2 (>3.0, ⩽5.0 cm)	144	10 (5, 8)	0.473
	3 (>5.0, ⩽7.0 cm)	90	13 (5, 21)	0.142
	4 (>7.0 cm)	109	11 (5, 24)	0.109
Adjuvant chemotherapy	No	207	10 (4, 18)	
	Yes	230	11.5 (5, 22)	0.065

Abbreviations: CPCC, chromatin CKAP2 (cytoskeleton-associated protein 2)-positive cell count; LN, lymph node; MD, moderately differentiated adenocarcinoma; PD, poorly differentiated adenocarcinoma; SRC, signet-ring cell carcinoma; WD, well-differentiated adenocarcinoma; WHO, World Health Organization. Significant values are shown in bold.

**Table 2:** Univariate and multivariate analyses of overall survival for each clinicopathological parameter and CPCC

		*Total*	
*Variable*	*Group*	*HR (95% CI)*	P^a^
Gender	Male	1	
	Female	0.75 (0.52–1.08)	0.119
Age, years	<50	1	(**<0.001**)
	50–59	0.92 (0.56–1.50)	0.726
	60–69	1.74 (1.14–2.67)	**0.011**
	⩾70	2.59 (1.57–4.28)	**<0.001**
Histology (WHO)	WD–MD	1	
	PD	1.89 (1.32–2.72)	**0.001**
	SRC	0.91 (0.52–1.59)	0.748
	Others	2.34 (1.26–4.33)	**0.007**
Lauren classification	Intestinal	1	
	Diffuse	1.12 (0.79–1.61)	0.508
	Mixed	1.07 (0.59–1.93)	0.828
	Unknown		
Depth of invasion	T1	1	(**<0.001**)
	T2	2.43 (1.13–5.24)	**0.023**
	T3	5.60 (3.47–10.36)	**<0.001**
	T4	11.75 (7.13–19.35)	**<0.001**
LN metastasis	N0	1	(**<0.001**)
	N1	2.79 (1.54–5.04)	**0.001**
	N2	4.43 (2.65–7.43)	**<0.001**
	N3	9.29 (6.11–14.12)	**<0.001**
Metastasis	No	1	
	Yes	7.94 (5.06–12.45)	**<0.001**
Tumor location^b^	Upper	1	
	Middle	1.09 (0.61–1.95)	0.773
	Lower	0.83 (0.50–1.38)	0.468
	Overlapping	3.52 (1.95–6.35)	**<0.001**
Tumor size	1 (⩽3.0 cm)	1	(**<0.001**)
	2 (>3.0, ⩽5.0 cm)	1.77 (0.99–3.17)	0.054
	3 (>5.0, ⩽7.0 cm)	2.34 (1.28–4.27)	**0.006**
	4 (>7.0 cm)	5.82 (3.37–10.03)	**<0.001**
Adjuvant chemotherapy	No	1	
	Yes	5.45 (3.65–8.13)	**<0.001**
CPCC (univariate)	Group 1 (⩽6, *N*=156)	1	(0.188)
	Group 2 (7–17, *N*=151)	0.94 (0.64–1.40)	0.765
	Group 3 (⩾18, *N*=130)	1.30 (0.89–1.89)	0.178
CPCC (multivariate)^c^	Group 1 (⩽6, *N*=156)	1	(0.620)
	Group 2 (7–17, *N*=151)	0.80 (0.52–1.23)	0.301
	Group 3 (⩾18, *N*=130)	0.90 (0.59–1.37)	0.610

Abbreviations: CI, confidence interval; CPCC, chromatin CKAP2 (cytoskeleton-associated protein 2)-positive cell count; HR, hazard regression; LN, lymph node; MD, moderately differentiated adenocarcinoma; PD, poorly differentiated adenocarcinoma; SRC, signet-ring cell carcinoma; WD, well-differentiated adenocarcinoma; WHO, World Health Organization.

a The *P*-value from the univariate or multivariate analyses is shown. The *P*-value for the linear trend is shown in parentheses.

b The tumor location was classified as follows: upper tumor, tumor located in the upper 1/3 of the stomach; middle tumor, tumor located in the middle 1/3 of the stomach; lower tumor, tumor located in the lower 1/3 of the stomach; overlapping tumor, tumor at the borderline between upper and middle tumors or between middle and lower tumors.

c Multivariate analysis with a Cox proportional hazard regression model was used with the co-variables of age, T stage, N stage, histology, tumor location, tumor size, and adjuvant chemotherapy. All patients included in the univariate analysis were also included in the multivariate analysis.

Significant values are shown in bold.

**Table 3:** Univariate and multivariate analyses of overall survival in various gastric cancer patient subgroups, according to CPCC

			*Univariate*		*Multivariate*^a^	
*Subgroup*	*Group*	N	*HR (95% CI)*	P	*HR (95% CI)*	P
Male	Group 1 (⩽6)	107	1		1	
	Group 2 (7–17)	103	0.95 (0.60–1.51)	0.835	0.71 (0.42–1.21)	0.206
	Group 3 (⩾18)	91	1.45 (0.93–2.25)	0.097	1.09 (0.66–1.83)	0.715
T1 or T2 male	Group 1 (⩽6)	62	1		1	
	Group 2 (7–17)	50	0.55 (0.17–1.77)	0.315	1.42 (0.38–5.33)	0.602
	Group 3 (⩾18)	46	2.36 (1.02–5.46)	**0.045**	3.05 (1.06–8.76)	**0.039**
T3 or T4 male	Group 1 (⩽6)	45	1		1	
	Group 2 (7–17)	53	0.83 (0.50–1.37)	0.461	0.75 (0.40–1.43)	0.386
	Group 3 (⩾18)	45	1.05 (0.62–1.77)	0.854	0.88 (0.46–1.66)	0.684
Female	Group 1 (⩽4)	50	1		1	
	Group 2 (5–13)	42	1.03 (0.50–2.14)	0.935	1.68 (0.59–4.81)	0.333
	Group 3 (⩾14)	44	0.75 (0.35–1.62)	0.467	0.88 (0.36–2.14)	0.773

Abbreviations: CI, confidence interval; CPCC, chromatin CKAP2 (cytoskeleton-associated protein 2)-positive cell count; HR, hazard regression.

a Multivariate analysis with a Cox proportional hazard regression model was used with the co-variables of age, T stage, N stage, histology, tumor location, tumor size, and adjuvant chemotherapy. All patients included in the univariate analysis were also included in the multivariate analysis.

Significant values are shown in bold.
