# Supplementary figures and images for: Clinical implications of proliferation activity in T1 or T2 male gastric cancer patients
Source: Exp Mol Med. 2015 Nov 6;47(11):e193–. doi: 10.1038/emm.2015.79 (PMC4673469; doi:10.1038/emm.2015.79)

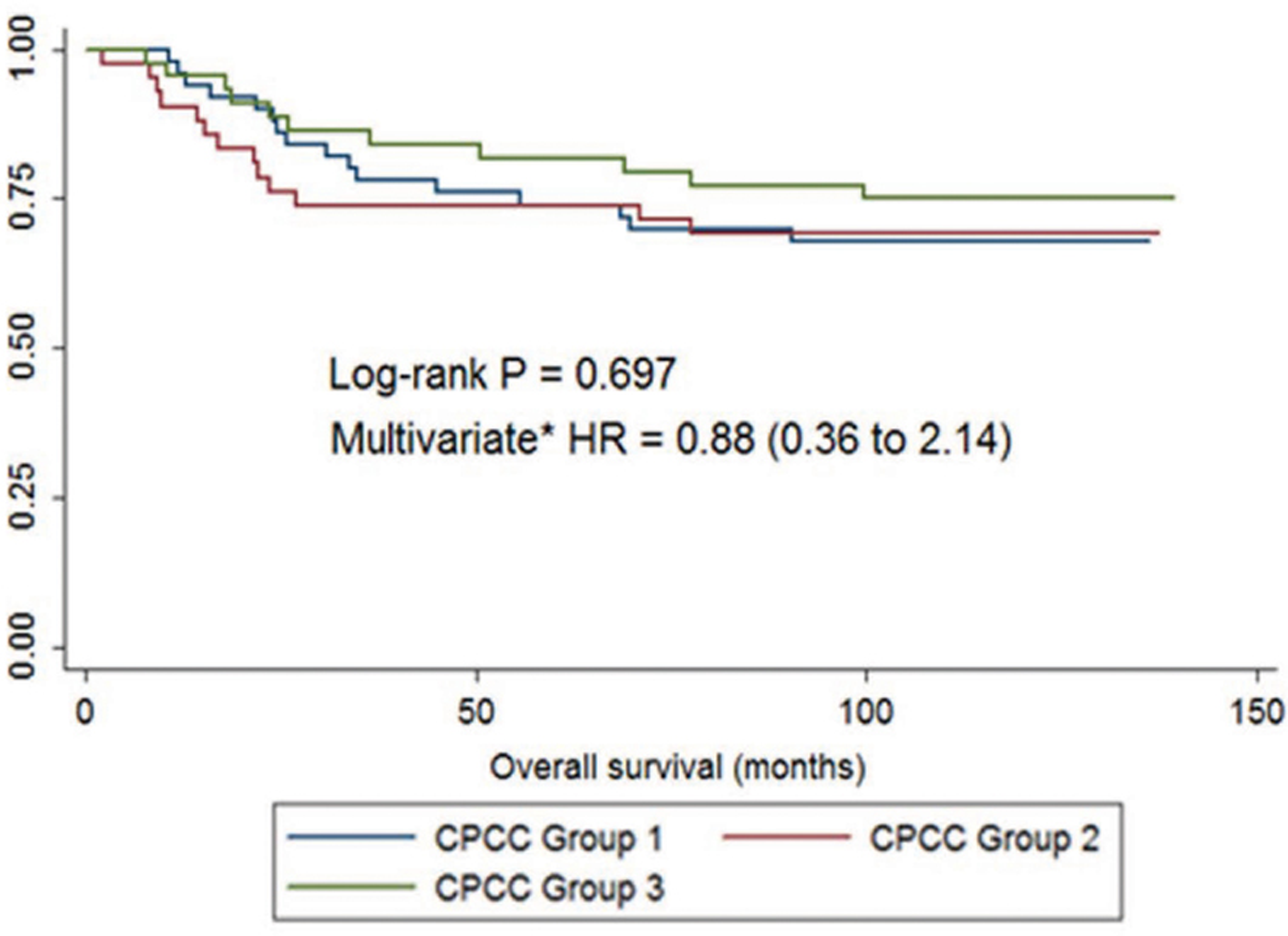

Supplement: Supplementary Figure 1 [file emm201579x1.tif]
