# Supplementary material for: Clinical implications of proliferation activity in T1 or T2 male gastric cancer patients
Source: Exp Mol Med. 2015 Nov 6;47(11):e193–. doi: 10.1038/emm.2015.79 (PMC4673469; doi:10.1038/emm.2015.79)
Supplement: Supplementary Information [file emm201579x2.docx]

**Figure S1. Kaplan-Meier plot of the CPCCs in the female gastric cancer.** The *P* value was determined by log-rank test. CPCC group 3 was compared with CPCC group 1 for HR and 95% CI by multivariate analysis. CPCC, chromatin CKAP2-positive cell count; Y-axis, survival probability
